# Evolution of Skin during Rehabilitation for Elephantiasis Using Intensive Treatment

**DOI:** 10.1155/2016/4305910

**Published:** 2016-11-24

**Authors:** Henrique Jose Pereira de Godoy, Ricardo Budtinger Filho, Maria de Fatima Guerreiro Godoy, José Maria Pereira de Godoy

**Affiliations:** ^1^Universidade Federal de Mato Grosso (UFMT), Cuiabá, MT, Brazil; ^2^Research Group of the Clínica Godoy, São José do Rio Preto, SP, Brazil; ^3^Postgraduation Course of the Medicine School in São José do Rio Preto (FAMERP) and Research Group of the Clínica Godoy, São Jose do Rio Preto, SP, Brazil; ^4^Research Group of Godoy Clinic, Cardiology and Cardiovascular Surgery Department of the Medicine School in São José do Rio Preto (FAMERP), São José do Rio Preto, SP, Brazil

## Abstract

The objective of this study is to describe the evolution of the skin during rehabilitation for elephantiasis using intensive treatment. We report on the case of a 55-year-old patient with a seven-year history of leg edema. The patient reported that it began with repeated outbreaks of erysipelas over several years. One leg evolved with significant edema leading to an inability to ambulate and for about one month the patient said that he could not get out of bed. Moreover the patient was obese weighing 130 kilos and with a BMI of 39. Intensive treatment was performed over three weeks resulting in a significant reduction in limb volume. The treatment consisted of Mechanical Lymphatic Therapy (RAGodoy), Cervical Lymphatic Stimulation (Godoy & Godoy technique), and a custom-made inelastic stocking of a grosgrain textile. What caught the attention during therapy were the open wounds resulting from fragmentation of the plaque as the edema reduced; the plaque was about 0.5 cm thick. As the treatment evolved the plaque disappeared and the wounds healed. The limb size decreased by more than 80% in three weeks after which the patient began to be treated in an outpatient setting with ambulation using a grosgrain stocking.

## 1. Introduction

Lymphedema is a chronic clinical sign, usually progressive, of the accumulation of macromolecules in the interstitial space leading to fluid retention in certain body parts, especially the extremities. It can be primary or secondary; primary lymphedema is defined by changes in the lymphatics at birth whereas secondary lymphedema is related to acquired changes [1].

Diagnosis is clinical but sometimes requires confirmation using supplementary exams. Its classification is divided into four clinical stages where 0 is subclinical, edema forms during the course of the day and disappears on resting in stage I, the patient has edema continuously even after resting for a week in stage II, and stage III is a deterioration of stage II where patients have major deformities [1, 2].

An association of different types of therapy is recommended; lymph drainage, compression therapy, and myolymphokinetic exercises constitute the basis of physical therapy [1]. In recent years Godoy & Godoy have developed new concepts of lymph drainage and new compression garments [2]. Moreover, an intensive approach had been developed which enables large volume reductions in a short period (around 50% of volume in five days) [2, 3]. The aim of this study is to describe the evolution of the skin during rehabilitation for elephantiasis and the difficulties encountered.

## 2. Case Report

We report on the case of a 55-year-old patient with a seven-year history of leg edema. The patient reported that it began with repeated outbreaks of erysipelas over several years for which he received treatment, but he did not remember which drugs were prescribed. One leg evolved with significant edema leading to an inability to ambulate and for about one month the patient said he could not get out of bed. Moreover the patient was obese weighing 130 kilos and with a BMI of 39. The diagnosis of lymphedema was based on the clinical history and a physical examination. The appearance of the lesion did not suggest a neoplasm and so intensive treatment was prescribed.

**Figure 1:**
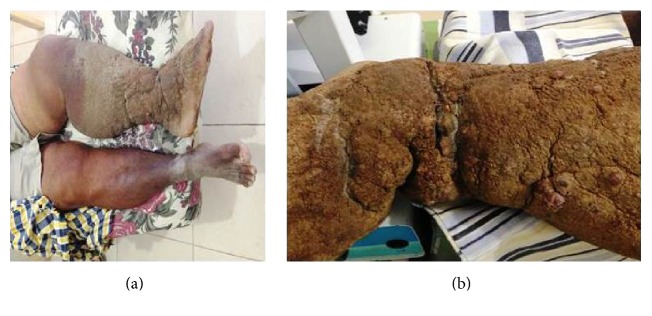
Skin plaque of severe lymphedema of the leg and foot with significant involvement of the ankle joint.

**Figure 2:**
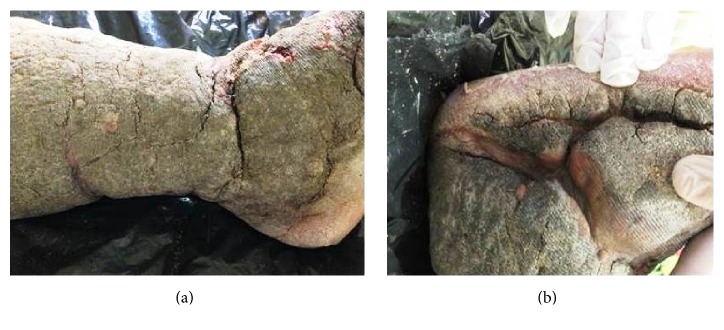
Evolution during therapy allowing the identification of grooves and the appearance of skin lesions.

**Figure 3:**
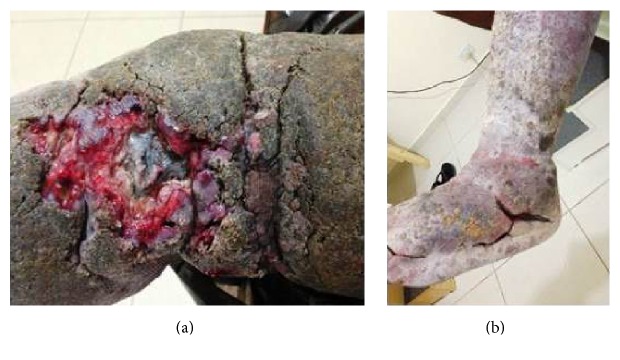
Plaque with a thickness of 0.5 to 1 cm thick which was eliminated with treatment.

**Figure 4:**
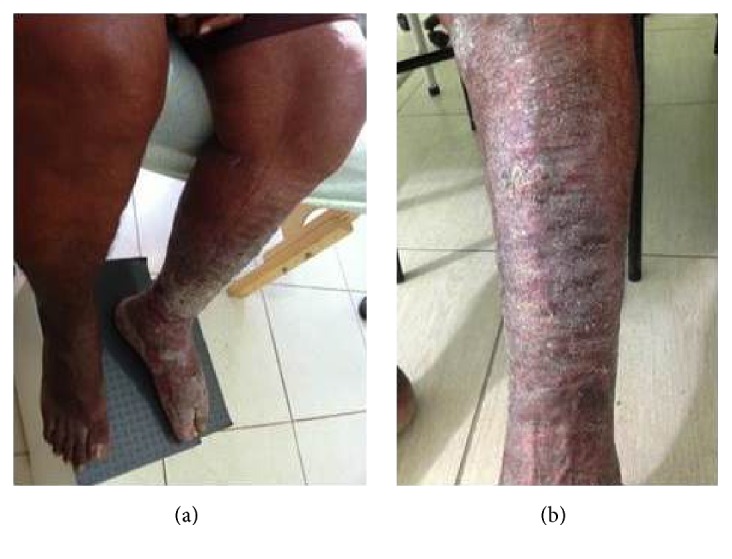
Evolution of the skin with functional recovery of the limb and disappearance of the plaque with treatment.

The most striking aspects of the physical examination of the patient were obesity and lymphedema; however the thickness of the plaque that covered the skin indicated the chronicity of the disease (Figures 1(a) and 1(b)). Intensive treatment was performed for three weeks in which time there was a significant reduction in limb volume. Treatment consisted of Mechanical Lymphatic Therapy (RAGodoy) [4] for eight hours per day associated with 15 minutes of Cervical Lymphatic Stimulation (Godoy & Godoy technique) [5] and a custom-made inelastic stocking of a grosgrain textile associated with an Unna boot. The stocking was used throughout treatment even at night. What caught the attention during treatment was the fragmentation of the plaque with open wounds as the edema was reduced; the plaque was about 0.5 cm thick (Figures 2(a), 2(b), and 3(a)). As the treatment evolved the wounds healed and the plaque disappeared (Figures 3(b), 4(a), and 4(b)). The use of moisturizers helped to remove the plaque. After a reduction of more than 80% in volume over three weeks the patient began to receive outpatient treatment using a grosgrain stocking and ambulation. After three months, the lesions were healed and almost all of the plaque had been removed.

## 3. Discussion

This study describes the rehabilitation of a patient with debilitating elephantiasis using intensive lymphedema treatment over three weeks followed by outpatient treatment. With therapy a severely handicapped patient was able to walk again and continue his treatment. There are no publications in the literature about the normalization or near normalization of the skin in patients with advanced grade II lymphedema or elephantiasis. Advanced degrees of lymphedema are characterized by significant fibrosis of the skin. Thus, this case report is an important contribution to medicine as it shows that severe lesions of the skin due to lymphedema can be reverted to almost clinically normal skin.

Lymphedema has three well-defined clinical stages: stages I, II, and III. Stage 0 or subclinical lymphedema was included in this classification to identify at-risk patients. The most commonly used therapeutic approach around the world is combinations of lymph drainage, exercises, restraint mechanisms, and hygienic care. The method used in the current case proposes a combination of therapies based on new concepts of manual and mechanical lymph drainage, restraint mechanisms. In the authors' experience this approach can result in normalization or near normalization of the limb size in all the different types of lymphedema including elephantiasis. This study clearly shows the radical change of the skin with this form of therapy leading to an almost normal appearance of the leg.

The evolution of skin with fragmentation of the plaque and formation of ulcers occurred as the size of the leg was reduced. In this phase, prophylactic measures using antibiotics were taken to protect against infection. The use of an Unna boot allowed the patient to diminish the limb volume to near normal size. The differential of this therapy is the reduction of edema, and as the limb volume reduced, the improvement in the skin becomes more evident. The elimination of the plaque was important as it allowed the appearance of normal skin.

The treatment of lymphedema mobilizes large amounts of macromolecules and fluids that are redistributed to other regions of the body, especially to normal areas [5]. Hyaluronic acid is one of the macromolecules in the interstitial space; its redistribution improves skin hydration as was reported by this patient.

The use of moisturizers facilitates the removal of the fragmented plaque thereby reducing the risk of skin injury. As the leg perimeter reduced, the size of the plaque also needed to decrease, but this was impossible due to its rigidity. Therefore the reduction in leg size caused the plaque to crack sometimes resulting in open wounds.

Surgery is becoming increasingly less indicated for cases of lymphedema. Thus lymph drainage was the basis of treatment in this case, showing how it can be useful to recover the skin.

## 4. Conclusion

Severe elephantiasis may progress with significant skin changes including thick plaque which hinders the treatment of lymphedema. However it is possible to clinically treat elephantiasis.
